# Biobased carbon content of resin extracted from polyethylene composite by carbon-14 concentration measurements using accelerator mass spectrometry

**DOI:** 10.1186/2193-1801-3-6

**Published:** 2014-01-03

**Authors:** Kazuhiro Taguchi, Masao Kunioka, Masahiro Funabashi, Fumi Ninomiya

**Affiliations:** Research Institute for Innovation in Sustainable Chemistry, National Institute of Advanced Industrial Science and Technology (AIST), Central 5, 1-1-1 Higashi, Tsukuba, Ibaraki, 305-8565 Japan

**Keywords:** Polyethylene, Biobased carbon content, Accelerator mass spectrometry, Radiocarbon, Additives

## Abstract

An estimation procedure for biobased carbon content of polyethylene composite was studied using carbon-14 (^14^C) concentration ratios as measured by accelerated mass spectrometry (AMS). Prior to the measurement, additives and fillers in composites should be removed because they often contain a large amount of biobased carbon and may shift the estimation. Samples of resin with purity suitable for measurement were isolated from composites with a Soxhlet extractor using heated cyclohexanone. After cooling of extraction solutions, the resin was recovered as a fine semi-crystalline precipitate, which was easily filtered. Recovery rates were almost identical (99%), even for low-density polyethylene and linear low-density polyethylene, which may have lower crystallinity. This procedure could provide a suitable approach for estimation of biobased carbon content by AMS on the basis of the standard ASTM D 6866. The biobased carbon content for resin extracted from polyethylene composites allow for the calculation of biosynthetic polymer content, which is an indicator of mass percentage of the biobased plastic resin in the composite.

## Background

**Figure 1:**
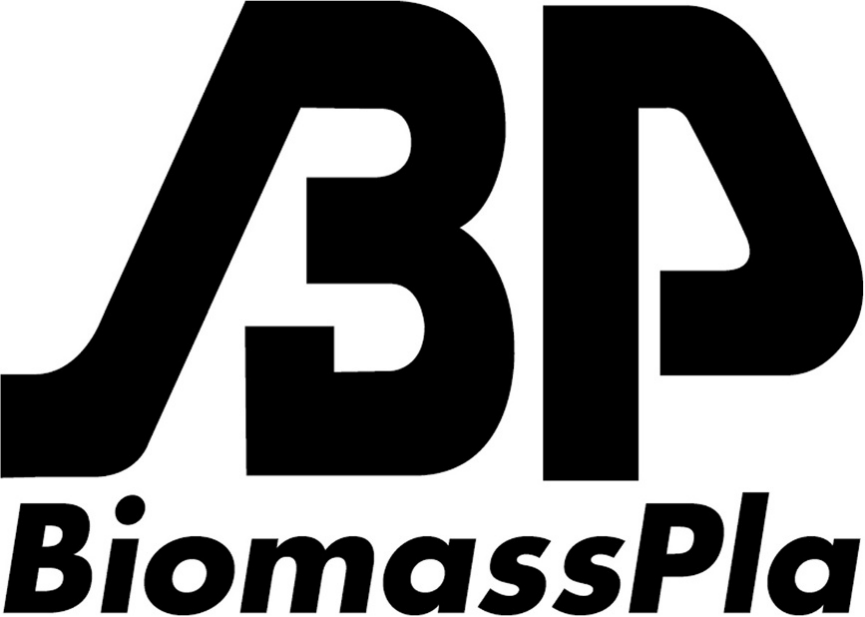
**Symbolic mark of biomass certification system.**

Biobased plastics such as poly(lactic acid) and poly(hydroxyl alkanoic acid) are already produced commercially and are steadily gaining in popularity with public awareness of the environment. Furthermore production of polyethylene and polypropylene, which are major thermoplastic resins, is now achieved from biomass resources (Morschbacker 2009; Peters et al. 2010; Takahashi et al. 2012). To be certain of purchasing biobased plastics, it should be confirmed and certified that they are actually produced from biomass, and, if they must be, how much biobased plastic is contained in the plastics. Products of biomass origin and products of petroleum origin are indistinguishable because they have the same physical and chemical properties if they have same molecular structure. Therefore, in an attempt to increase general consumer knowledge and promote biobased plastics, the Japan Bioplastics Association (JBPA) (Japan Bioplastics Association 2013) is managing the “BiomassPla” mark certification system as an identification system for products of biomass origin. Under this system, products that meet the stipulated standards are certified as BiomassPla and are permitted to use the “BiomassPla” logo shown in Figure 1. The degree of biobased synthetic polymer in a product shall be a plastics product of 25.0 wt% or more in one of the authentication conditions of the aforementioned system. The degree of biobased synthetic polymer is the ratio of the biomass origin resin to the plastic product (Table 1).

**Table 1:** **Bio-polyethylene and various fillers and additives in plastic products**

	Resins	Solvent insoluble additives (Fillers)	Solvent soluble additives
Bio-based	Bio-polyethylene (**a**)	Starch, Cellulose, Graphite (**c**)	UV absorbent, Flame retarder (DBDPE) (**e**)
Petroleum-based	(**b**)	Calcium carbonate (**d**)	(**f**)

(Resin content) = ((**a**) + (**b**))/(Composite).

(Biobased synthetic polymer content) = (**a**)/(Composite).

The biobased carbon ratios of plastics can be estimated by the ratio of ^14^C to ^12^C measured by accelerator mass spectrometry (AMS) conforming to the standard ASTM D 6866 “Standard Test Methods for Determining the Biobased Content of Natural Range Materials Using Radiocarbon and Isotope Ratio Mass Spectrometry Analysis.” The principle of this method using ^14^C is based on a dating measurement for historical materials in archeology (Jull and Burr 2006; Currie 2004). ^14^C is a radioisotope of carbon atoms with a half-life of 5730 years. ^14^C atoms are continuously generated from ^14^ N atoms due to their interaction with cosmic radiation in the modern atmosphere. The ratio of ^14^C to ^12^C in modern air is constant at approximately 1 × 10^-12^ in spite of the period. Plants absorb carbon dioxide in the atmosphere and incorporate it in their structure by photosynthesis. The ratio of ^14^C to ^12^C in a plant is 1 × 10^-12^ immediately after photosynthesis. The ^14^C in plant materials gradually decays into ^14^ N. The number of ^14^C atoms continuously decreases and becomes half after 5730 years. Therefore, the age of materials including carbon atoms can be estimated using the ratio of the number of ^14^C atoms to that of ^12^C atoms and the half-life of ^14^C. The ratio of ^14^C to ^12^C can be measured by AMS, although this ratio is as low as 1 × 10^-12^. The standard year is defined as 1950 according to the formulas for radiometric dating in ASTM D 6866–08. In ASTM D 6866–08, formulas for radiometric dating are applied to the determination of the biobased carbon content. A percent modern carbon (pMC) value can be estimated by comparing the measured ratio of ^14^C to ^12^C, and the standard ratio of ^14^C to ^12^C determined from the appropriate primary reference (oxalic acid) of SRM 4990c supplied by the National Institute of Standards and Technology (NIST), USA (SRM 2013). Theoretically, biobased carbon ratios for petroleum-based materials are estimated at 0%, and for biobased materials at 100%. Our previous reports (Funabashi et al. 2009; Onishi et al. 2010; Tachibana et al. 2010) described estimation of biobased carbon ratios for various polymeric composites with additives and fillers, and discussed repeatability and accuracy of this evaluation method. For reliable estimation of the ratios, we devised pretreatments for AMS samples such as lower-temperature oxidation and reaction by phosphoric acid.

**Figure 2:**
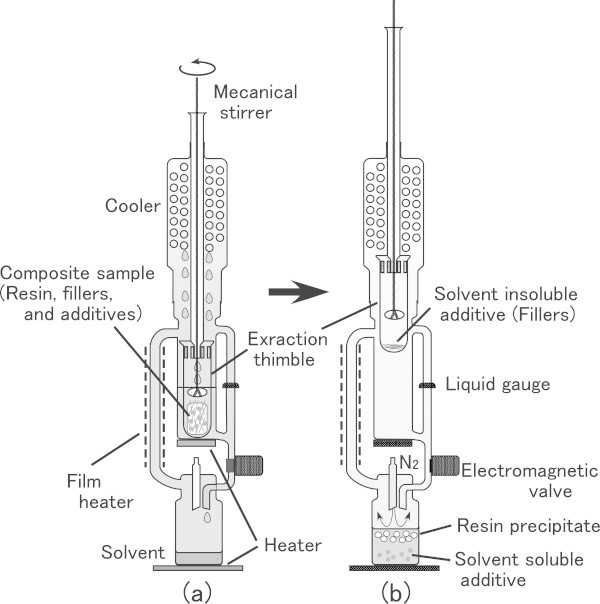
**Separation of the resin component from polyethylene composites by a Soxhlet extractor.** **(a)** Extraction with cyclohexanone in progress, and **(b)** precipitation of resin after extraction.

This report describes a pretreatment for an AMS sample of polyethylene products with additives and fillers. Before evaluation of biobased carbon content in bio-plastic products by AMS, isolation of resin is necessary to confirm the presence of biobased polyethylene. For isolation of resins we used a Soxhlet extractor, which is a general apparatus for the separation of solvent soluble components from solid materials and has also been used in polymer science. For example, it has been used for the separation of additives from polyolefins using methylene dichloride (El Mansouri et al. 1998), separation of oligomers from polypropylene using n-heptane (1999), and removal of insoluble parts of cross-linked polyethylene using xylene (Elzubair et al. 2003). We employed a commercial instrument that allows quick extraction at an elevated temperature by heating an extraction chamber of the instrument (Figure 2). In addition to resin isolation as a pretreatment, we considered evaluating the resin content of polyethylene composites. There is no useful method for measuring the content of resin components in polyethylene products because of the variety of chemical and physical properties of plastic products. Linear low-density polyethylene (LLDPE) and low-density polyethylene (LDPE) have side chains and branches coming off the main chains. LLDPE and LDPE are different in crystallinity as compared to high-density polyethylene (HDPE), which is composed of a fundamental structure of methylene chains, resulting in substantially different thermal properties (melting point) and X-ray diffraction patterns (Zhu et al. 1999; Mirabella and Bafna 2002; Perez et al. 2000). Therefore, these methods are not useful. Even FTIR measurement, which is the most effective and easiest-to-use analytical method for polymeric materials, can provide unclear results. This is because the presence of branches or side chains on polymer main chains and the lack of uniformity of samples may deform the intensity of absorption bands of the fundamental structures (methylene chains) of polyethylene (Koenig 1992; Hagemann et al. 1989). Furthermore, additives and fillers may interfere with the measurement due to overlapping spectra. As a result, we concluded that the amount of resin recovered from an extraction solution should be viewed as the most sensible value for resin content of plastic products, regardless of incomplete precipitation from the extraction solution and handling loss of recovered resins.

The procedure for polyethylene isolation by a Soxhlet extractor in this study was based on a simple principle: difference in solubility behavior for each component in the composites. That is, polyethylene is soluble in a hydrophobic solvent at an elevated temperature and near insoluble at room temperature (Brandrup and Immergut 1966; Barton 1975). After cooling the extraction solution, resin components should form a dense precipitate and be successfully recovered by filtration of the entire extraction solution. Fillers such as graphite, calcium carbonate, starch, and cellulose are insoluble in hydrophobic solvents and remain in an extraction thimble throughout the operation. Organic additives such as antioxidants, UV-absorbents, and flame-retardants, which are mostly low molecular weight substances, have good solubility in organic solvents even at room temperature (Bolgar et al. 2008; Tolinski 2009). Various hydrophobic chemicals are known as good solvents for polyethylene at elevated temperature: hydrocarbons such as xylene and dodecane, and chlorinated hydrocarbons such as 1,2-dichlorobenzene and 1,2,4-trichlorobenzene. However, in this study we used cyclohexanone, which is typically not a good solvent for polyolefins due to a polar carbonyl function on the molecule, to improve resin recovery from the extraction solution. By cooling the solution to room temperature, polyethylene can form a precipitate, whereas hydrophobic organic additives remain in the extraction solution. Filtering the floating polyethylene precipitate and rinsing with a volatile solvent can yield a good sample with purity suitable for AMS measurement, and at the same time, can provide an estimate of the amount of resin content of composites.

**Scheme 1:**
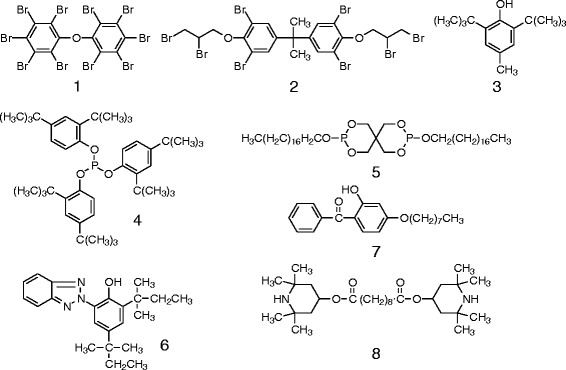
**Additives examined in this study.**

Various kinds of additives, including antioxidants, UV-absorbents, nucleation agents, flame-retardants, and fillers, are usually applied to polyethylene products in combination. Extensive testing of composites covering a variety of commercial additives is impractical. Therefore, we chose some fillers (graphite and calcium carbonate), which contain petroleum-based carbon, and others such as pulverized oyster shell (Gofun, where calcium carbonate is a major ingredient), starch, and cellulose, which may often be used in high quantities in polyethylene composites, and may drastically increase estimations of biobased carbon content owing to the bio-origin of carbon. Soluble additives used in commercial polyethylene products are too numerous to count. However, a limited number of compounds may be sufficient for testing because almost all of them have common characteristic properties due to the hydrophobic functional groups needed to retain compatibility with the hydrophobic properties of the resin (see Scheme 1). In spite of the differences in their chemical skeletons and functions, they could be classified together based on their solubility in hydrophobic solvents. Therefore, we tested a flame retardant (decabromodiphenyl ether, DBDPE) because large amounts of flame retardants are usually added (for instance 40 wt%), compared to small amounts of other additives (less than 1%). Another reason the flame-retardant was chosen was due to the fact that this compound has UV absorption properties and could be readily detected.

In this study we planned to confirm whether the isolation procedure of polyethylene proceeds successfully enough to be adapted as part of a standard method for estimating biobased carbon content of industrial products. An important issue is whether a hot solution of composite provides a dense precipitate that could be readily separated from the extraction solution. Physical properties of the precipitates were studied by scanning-electron microscope observation, differential scanning calorimetry (DSC), X-ray diffraction, and UV-visible measurements to confirm crystallinity of the precipitates and adequate removal of the additive from the composites.

## Materials and methods

### Materials

Materials in this study were purchased from the following companies: biobased high-density polyethylene (bHDPE, 0.956 g/cm^3^, MFR: 2.2 g/10 min (190°C/2.16 kg)) and biobased linear low-density polyethylene (bLLDPE, 0.916 g/cm^3^, MFR: 1.0 g/10 min (190°C/2.16 kg), co-monomer: 1-butene), Toyota Tsusho Co.; petroleum-based low-density polyethylene (pLDPE, 0.92 g/cm^3^, Mw 50,000, Tm 107–135°C), Scientific Polymer Products; petroleum-based high-density polyethylene (pHDPE) and graphite (grain size < 20 μm), Sigma-Aldrich Co.; calcium carbonate and cornstarch, Wako Pure Chemical Industries; decabromodiphenyl ether (DBDPE), Tokyo Chemical Co.; cellulose microcrystalline powder (Avicel® PH-M25, average size 25 μm), Asahi Chemical Industry Co., Japan. Except for cornstarch and cellulose, all fillers and additives were petroleum-based chemicals. Other chemicals were reagent grade and used without further purification. Extraction thimbles were products of Toyo Roshi Kaisha (Advantec, No. 89S, PTFE/quartz fiber, outer diameter of 25 mm, length of 90 mm).

### Soxhlet extraction instrument

A Soxhlet extractor (Büchi Co. B-811) was used as supplied by the manufacturer. However, to shorten dissolution time of polymers, a stirrer bar (15 mm long) was inserted into an extraction thimble. The bar was linked with a motor using a stainless steal wire through a small hole in the cooling tower of the instrument. A glass tube that supplied hot vapor from the solvent reservoir at the bottom to an extraction chamber was wrapped with a film heater to facilitate hot vapor supply. Nitrogen gas was continuously added into the instrument chamber to avoid oxidation of resins.

### Preparation of polyethylene composites

Before preparation of polyethylene composites, the fillers (graphite, calcium carbonate, Gofun, cornstarch, and cellulose) were dried at 120°C under reduced pressure until constant weights were obtained. Composite sheets with a thickness of 0.50 mm were prepared according to the previous report (Onishi et al. 2010). Polyethylene fine powder with a particle size less than 125 μm and an additive or fillers were mixed using a mortar and pestle. The resulting mixture was heated to 200°C at 20 MPa for 5 min in a stainless steel mold, and was gradually cooled by standing at room temperature.

### Isolation of polyethylene resin from composites

The procedure of extraction and isolation of polyethylene resins from the composites was carried out as follows. A sample of 500 mg of a composite sheet (in small pieces of approximately 3 mm square) was placed in an extraction thimble, and 125 mL of a solvent was placed in a solvent reservoir. The instrument was operated under Soxhlet Warm mode, which is the same as the standard operation of the traditional Soxhlet extractor except for heating the extraction chamber to facilitate extraction. Extraction was carried out for 3 h under a nitrogen atmosphere to avoid oxidation of components, and stirring was used at a rotation rate of 100 rpm in an extraction thimble to facilitate solubilization of the resins. Polyethylene precipitates formed in 30 min as the extractions cooled. Formation of precipitate from solution was rapid (in one min if the solution was cooled with running water). As a precaution solutions were allowed to stand for 3 hours, and the precipitates were subsequently recovered by filtration using a polytetrafluoroethylene (PTFE) membrane filter (Millipore Co., pore size: 0.45 μm). The precipitates were rinsed with the solvent for extraction, then with a volatile solvent (ethanol). The samples for scanning electron microscope observation, X-ray diffraction, and thermal analysis were dried under vacuum for 24 hr without heating to avoid altering the surface morphology and the crystallinity of the samples. For other purposes (weighing of resin recovery, UV-visible, AMS measurements, and ICP analysis) drying was conducted at 60°C to shorten the drying time.

### Recovery of fillers from composite

After the extraction operation the wet extraction thimbles were dried under vacuum at 80°C for 1 hr. Based on the weights of an extraction thimble before and after extraction, complete extraction was confirmed and the recovery rate of fillers from composites was calculated.

### Observation of polyethylene precipitate on scanning electron microscope (SEM)

Polyethylene precipitates were carefully transferred on an adhesive tape on a platform for SEM observation to avoid deformation of the specimens. Platinum deposition (4 nm thick) on the surface of the precipitates was conducted prior to observation using a field emission-scanning electron microscope (Hitachi High-Technologies Co., S-4300).

### Preparation of films for measurement of UV-visible spectra

Polyethylene films for measuring transmission of UV-visible spectra were prepared from polyethylene composites and their recovered precipitates by extraction. The samples were placed between a pair of glass plates, and heated under a nitrogen atmosphere at 160°C for 15 min using a pair of stainless steel thickness gauges to prepare films with a thickness of 0.1 mm. UV-visible spectra of the films and a chloroform solution (49 mg/L) of decabromodiphenyl ether as a reference were recorded at a scanning rate of 120 nm/min and a slit width of 2 nm on a spectrophotometer (Shimadzu Co. U-3000).

### Wide-angle X-ray scattering (WAXS)

X-ray scattering of polyethylene samples was recorded on a Rigaku Miniflex II diffractometer with CuKα-Ni-filter radiation. Plates of pure polyethylene with high crystallinity were used as a reference (24 mm in diameter and 2 mm in thickness); these were prepared on aluminum pans by heating at 150°C for 15 min and pressing the resin surfaces with a glass plate. After cooling at room temperature the resin plate was annealed at 100°C for 24 hr. The scan rate was 2°θ/min. WAXS patterns of polyethylene precipitates were obtained in the same way, by preparing the samples in the same aluminum pans in a way that was as compact as possible.

### Differential scanning calorimetry (DSC)

Melting properties of polyethylene samples were measured on a conventional DSC instrument (DSC 7020, Hitachi High-Tech Science Co, the former Seiko Instrument Inc.). Two scanning cycles of heating and cooling were carried out under a nitrogen gas atmosphere between −30°C and 150°C at a scan rate of 10°C/min.

### Analysis of calcium by radiofrequency inductively coupled plasma (ICP)

The precipitates obtained from the Soxhlet extractor using 500 mg of composite containing calcium carbonate or Gofun were moistened in 25 mL of acetone followed by 25 mL of 1% nitric acid. The mixture was agitated in a glass vessel using an ultrasonic bath for 3 hr. After the resin powder was filtered out, acetone in the filtrate was evaporated under reduced pressure. The resulting solution was diluted to 100 mL with 1% nitric acid and analyzed using an analytical instrument for radiofrequency inductively coupled plasma (ULTIMA2, HORIBA Ltd., the former Jobin Yvon S.A.S.). An amount of 500 mg of calcium carbonate or oyster shell (Gofun) powder was placed in an extraction thimble and treated with the common extraction operation. The extraction solvent in the reservoir was removed under reduced pressure until dry. The residue was added to 25 mL of 1% nitric acid and 25 mL of chloroform, and was shaken using a separable funnel to remove hydrophobic impurities derived from the extraction solvent. After the remaining chloroform, which was dissolved in the aqueous layer, was removed under reduced pressure, the aqueous solution was diluted with 1% nitric acid to 100 mL volume and analyzed using the ICP instrument.

## Results and discussion

The Soxhlet extractor is a sophisticated instrument for lab work, and the extraction of resin proceeded smoothly once a heating program was properly set with reference to an instruction provided by Büchi Co. In a preliminary experiment using resin pellets, completion of resin extraction could be confirmed via weight change of the extraction thimble before and after the operation. This was due to the low hygroscopicity of the extraction thimble; a small amount of remaining resin in the extraction thimble could be confidently detected (the weight increase of the extraction thimble was 0.21 wt%, or approximately 14 mg, when the extraction thimble was dried under vacuum at 60°C and exposed to air with 53% relative humidity). Solvent selection was generally the most important factor in extraction experiments. We tested several commercially available solvents that had proper solvency and boiling points (bp) for the resins. The dissolution rate of resins and the heating capacity of the instrument restricted the boiling point of solvents to around 150°C. The candidates included good solvents for polyolefins, such as o-xylene (bp 144°C) and 2-chlorotoluene (bp 159°C), and also unsuitable solvents including ketones and esters, such as cyclohexanone (bp 155°C), 2-heptanone (bp 151°C), 5-methyl-3-heptanone (bp 157°C), and amyl acetate (bp 148°C). In preliminary experiments for dissolution of polyolefins in test tubes, unsuitable solvents showed longer dissolution times for resins in comparison with good solvents. The extraction time for resins using the Soxhlet extractor was as long as expected. Extraction was completed using o-xylene for 1 h and cyclohexanone for 3 hr. Though a good solvent, o-xylene, was better in terms of extraction time, we ultimately used cyclohexanone because of the recovery rate of resins from extraction solvents and its wider commercial availability among several unsuitable solvents. Good solvents, such as o-xylene, sometimes produced swelling precipitates that were inconvenient for filtration of the extraction solution. This situation was not a serious issue for polyethylene resins, but it did pose a problem for other polyolefins, such as polypropylene and poly(1-butene). We preferred a common set of extraction conditions that would be applicable to a wide range of polyolefins. We plan to report elsewhere on the extraction experiments of polypropylene resins, including copolymers of propylene and ethylene, using good and unsuitable solvents.

**Figure 3:**
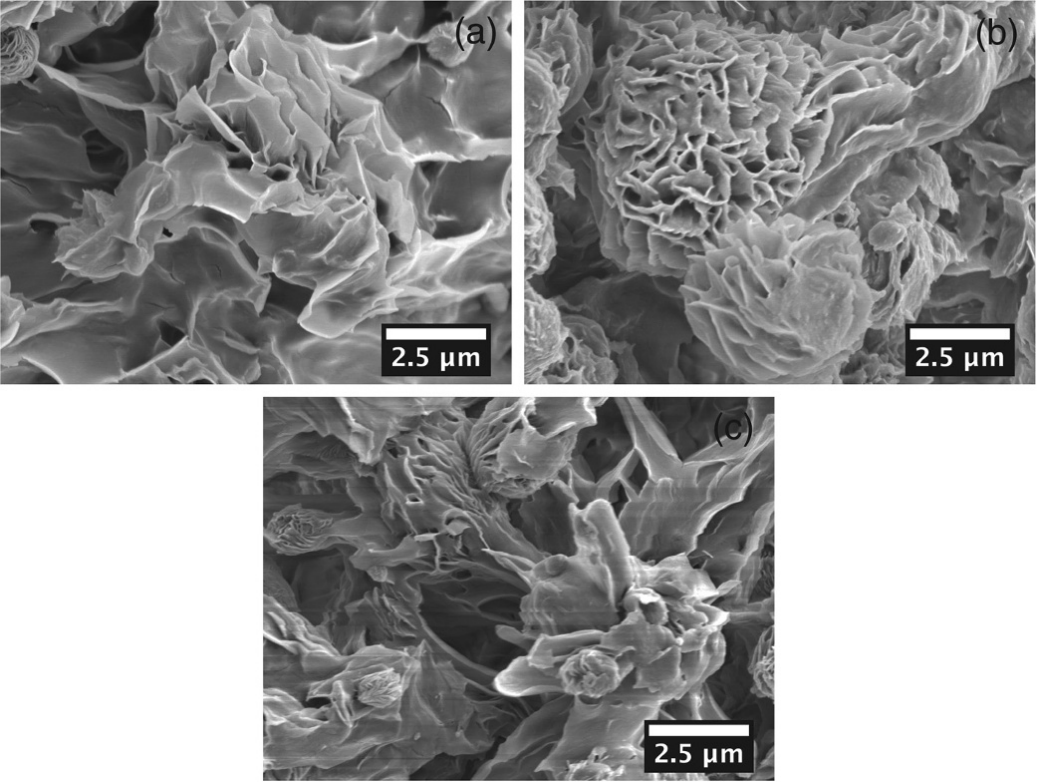
**Scanning electron microscope images of recovered precipitates prepared from 1% cyclohexanone solutions of polyethylene resins.** bHDPE **(a)**, bLLDPE **(b)**, and pLDPE **(c)**.

When heated extraction solutions were cooled to room temperature, the solubility of polyethylene decreased resulting in white precipitates of resin in 30 min. The precipitates were recovered on a porous PTFE membrane by filtration, followed by an ethanol rinse to shorten the drying time. bLLDPE and pLDPE, which have side chains or branching on main chains (making them problematic to crystallize), also formed the same bulky-looking but dense precipitates as bHDPE. As shown in Figure 3, scanning electron microscope observation showed that these precipitates were coarse-surfaced particles.

**Figure 4:**
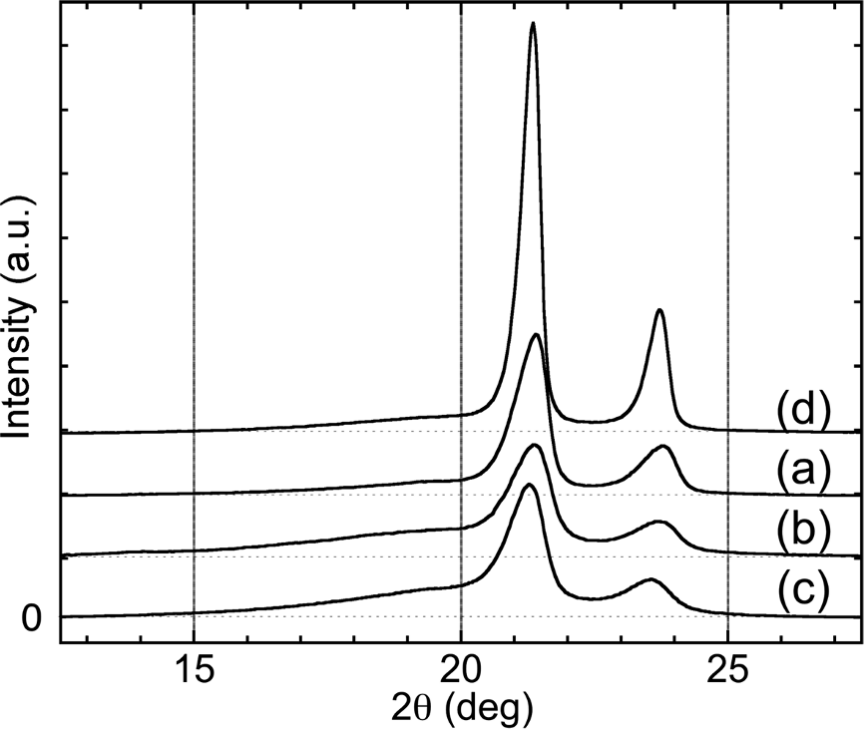
**WAXS patterns for precipitates.** bHDPE **(a)** bLLDPPE **(b)**, pLDPE **(c)**, and a plate of bHDPE prepared by annealing at 100°C for 24 h **(d)**.

X-ray diffraction of the precipitates obtained from polyethylene resins showed an overlap between the crystalline and amorphous patterns indicating that the precipitate was a semi-crystalline polyethylene, as shown in Figure 4a. The peaks were broader than that of a resin plate prepared by annealing at 100°C (Figure 4d). Even when the test tube was cooled rapidly in an ice-water bath, the hot solution produced dense precipitates instead of a swelling gel. Therefore, it is not necessary to control the cooling condition of the hot extraction solution to recover polyethylene precipitates. The hot solutions of composites with additives also formed dense precipitates, which could be just as readily separated as pure propylene resins.

**Figure 5:**
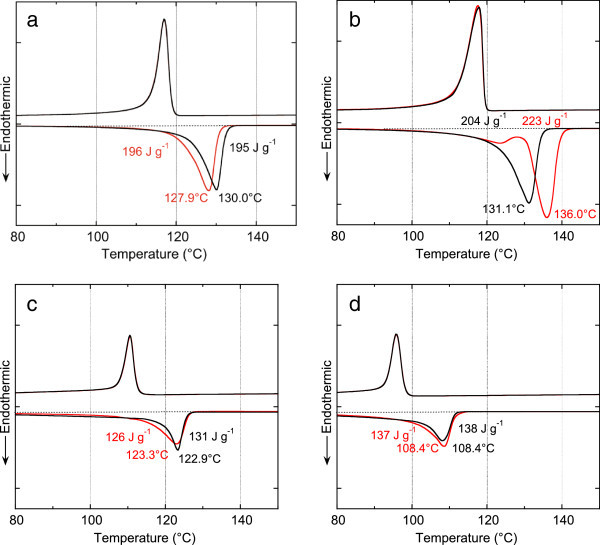
**Differential scanning calorimetry of polyethylene samples. (a)** precipitates of bHDPE, **(b)** bHDPE annealed at 100°C, **(c)** precipitates of bLLDPE, and **(d)** precipitates of pLDPE. Red line: first scanning. Black line: second scanning.

Melting properties observed by DSC were an indicator of crystallinity of the precipitates. An endothermic peak for bHDPE precipitates appeared at slightly lower temperature (127.9°C) during the first heating compared to the second heating (130.0°C) (Figure 5a). A substantial difference in enthalpy change between the first and second scans (196 J g^-1^, 195 J g^-1^, respectively) was not observed. The precipitates could be considered to have a moderate level of crystallinity when melting points and enthalpy changes are compared with those of resin annealed at 100°C (136.0°C, 223 J g^-1^) (Figure 5b). However, the thermogram and the XRD results indicated that the precipitates have good crystallinity.

Precipitates from bLLDPE showed a melting peak at a lower temperature and with lower enthalpy change (123.3°C, 126 J g^-1^) than those of bHDPE, as expected from chemical structures with side chains derived from a co-monomer (1-butene) (Figure 5c). Precipitates from pLDPE showed a melting peak at a lower temperature and lower enthalpy change (108.4°C, 137 J g^-1^), as expected from branching of main chains (Figure 5d). These thermal properties may be generally observed in polymeric materials where crystallization is hindered by branching or side chains. It is worth noting that the thermogram from the first scan was almost the same as the second scan. This indicates that the precipitates from extraction solutions hold almost the same physical properties as the bulk materials.

We concluded that the recovery rate of resins from composites accounts for the content of resin within composites. This assumption was based on the quantitative recovery of resins from the extraction solutions (Table 2). Excellent recovery rates were observed in spite of losses encountered during handling of precipitates for bHDPE (Entry 1, 98.8%), and further, for bLLDPE (Entry 5, 99.6%) and pLDPE (Entry 11, 98.9%). Low recovery rates for bLLDPE and pLDPE were worrisome because of the intrinsic low regularity of polymer chains. Crystalline properties of the polymers and prompt crystallization may explain the high recovery rate of resins.

**Table 2:** **Recovery rates of resins and fillers obtained by the Soxhlet extraction experiments of pure resins and composites**

Entry	Sample (w/w)	Recovery of resin (%) ^a^	Recovery of filler (additive) (%) ^a^	Total (%) ^b^
1	bHDPE	98.8 ± 0.4	(-0.1 ± 0.2)	(98.7 ± 0.3)
2	bHDPE/Graphite (75/25)	74.2 ± 0.9	25.1 ± 0.7	99.0 ± 1.0
3	bHDPE/CaCO_3_ (75/25)	73.6 ± 1.7	25.5 ± 3.0	99.1 ± 1.7
4	bHDPE/DBDPE (75/25)	73.7 ± 0.6	0.1 ± 0.1	73.8 ± 0.7
5	bLLDPE	99.6 ± 0.4	(0.2 ± 0.3)	(99.8 ± 0.1)
6	bLLDPE/Graphite (75/25)	74.0 ± 1.0	24.1 ± 0.6	98.1 ± 1.5
7	bLLDPE/CaCO_3_ (75/25)	73.6 ± 0.4	23.9 ± 0.3	97.5 ± 0.3
8	bLLDPE/DBDPE (75/25)	75.2 ± 0.4	−1.0 ± 0.1	74.2 ± 0.9
9	pHDPE/Starch (75/25)	74.9 ± 0.1	25.0 ± 0.1	99.9 ± 0.1
10	pHDPE/Cellulose (75/25)	74.6 ± 0.8	24.8 ± 0.8	99.5 ± 0.7
11	pLDPE	98.9 ± 0.4	(0.9 ± 0.5)	(99.7 ± 0.4)

^a^The recovery rate was calculated based on the total weight of the composite.

^b^Total weight of the recovered resin and the filler collected on extraction thimbles.

To quantitatively understand the removal of fillers from composites, polyethylene composites containing graphite, calcium carbonate, starch, and cellulose, were used for an extraction experiment, and the separation of the components was confirmed by the amount of recovered resins from extraction solutions and remaining fillers on the extraction thimbles. Recovery rates for resins from six composites (Entry 2, 3, 6, 7, 9, and 10) ranged from 73.6% to 75.2%, and the recovery rates for the remaining fillers ranged from 23.9% to 25.5% (Recovery rates used in this paper were calculated based on the total sample weight of the composites. The theoretical maximum recovery rates of resin and filler in this paper are 75% and 25%, respectively). These results were almost equivalent to the component percentage of the composites, and could satisfy our protocol when handling loss of fine precipitates and uneven quality of the composites are taken into account. In the case of the composites containing solvent-soluble additives (DBDPE, Entry 4 and Entry 10), the recovery rate of resins (Entry 4, 73.7% and Entry 8, 75.2%, respectively) were the same as the composites containing graphite or calcium carbonate, although the recovery rates of the additives were apparently null. This indicates that the additives that dissolved at elevated temperatures still remained in the extraction solution at room temperature and were not incorporated into the resin precipitates. From the composites containing starch or cellulose, additives, which may intentionally be added in order to raise the apparent biobased carbon content of plastic products, polyethylene resins were successfully isolated at the theoretical recovery rates (Entry 9, 74.9% and Entry 10, 74.6%).

The isolation of resins from the composites containing graphite or calcium carbonate, which do not contain biobased carbon, was confirmed on the basis of biobased carbon content measured by AMS. The biobased carbon content of the recovered precipitates from composites of bHDPE (Table 3, Entry 2, 97.7%, Entry 3, 98.1%, and Entry 4, 100.2%) were comparable to that of the original resin (97.7%) indicating effective elimination of the fillers from the composites. In case of composites of bLLDPE, clear isolation of resins was also confirmed: biobased carbon content for precipitates were measured at 87.4% (Entry 6) and 88.1% (Entry 7), whereas the original composite contained 88.4% (Entry 5). AMS results also affirmed that extraction of the composites containing the solvent-soluble additive DBDPE (Entry 4, 100.2% and Entry 8, 89.0%) yielded resin precipitates that were not contaminated with additive.

**Table 3:** **Bio-based carbon content of polyethylene resins and recovered precipitates measured by AMS**

Entry	Sample (w/w)	Measured (%)	Calculated for entire composite (%) ^b^
1	bHDPE	97.7 ± 0.3, 96^a^	
2	bHDPE/Graphite (75/25)	97.7 ± 0.2	70.3
3	bHDPE/CaCO_3_ (75/25)	98.1 ± 0.2	93.3
4	bHDPE/DBDPE (75/25)	100.2 ± 0.3	92.3
5	bLLDPE	88.4 ± 0.3, 87^a^	
6	bLLDPE/Graphite (75/25)	87.4 ± 0.3	63.6
7	bLLDPE/CaCO_3_ (75/25)	88.1 ± 0.2	84.5
8	bLLDPE/DBDPE (75/25)	89.0 ± 0.3	83.5

^a^Bio-based carbon content listed by the supplier.

^b^Bio-based carbon content calculated for composites based on mixing ratios of resins to fillers (additives).

**Figure 6:**
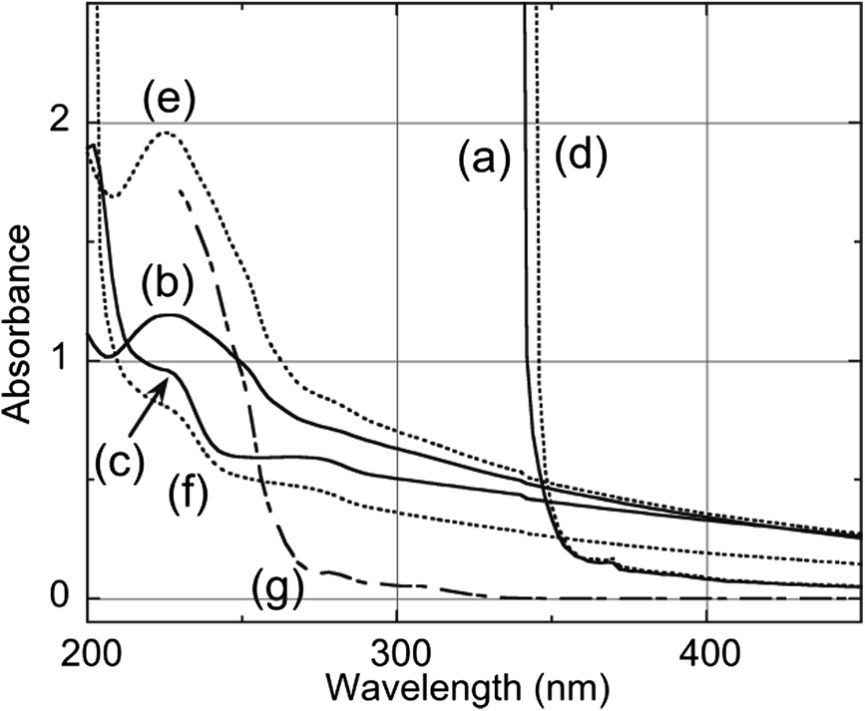
**UV-visible spectra of films prepared from composites (resin/additive, 75/25), the corresponding recovered precipitates, and original resins.** Composite of bHDPE/DBDPE **(a)**, precipitate from bHDPE/DBDPE **(b)**, bHDPE **(c)**, composite of bLLDPE/DBDPE **(d)**, precipitate from bLLDPE/DBDPE **(e)**, bLLDPE **(f)**, and chloroform solution of DBDPE **(g)**.

Figure 6 shows the UV-visible spectra of thin composite films of bHDPE and bLLDPE containing DBDPE (75/25) and the films prepared from the corresponding precipitates (Entries 4 and 8 in Table 2). The composite films of bHDPE (Figure 6a) and bLLDPE (d) showed a strong absorption (and scattering) in the ultraviolet region due to the aromatic skeleton of the additive molecule. On the other hand, the films from precipitates showed weak absorption bands and backgrounds with light scattering (b and e). A decrease in the absorption bands for the composites of the organic additive indicated the efficiency of the isolation process. Ratios of the additive to resins, calculated from a chloroform reference solution of the additive (g), were small (0.02 wt% for bHDPE (Entry 4) and 0.04 wt% for bLLDPE (Entry 8)). These findings suggest that the formation of pure precipitates lacking any accompanying additive may depend on prompt crystallization of polymers from the solutions and a high degree of crystallinity of the polymer.

Our results indicate that the extraction solvent should have enough solvency for additives at room temperature. Solubility tests were conducted for typical additives: decabromodiphenyl ether (flame retarder, DBDPE) (**1**), 2,2-Bis[3,5-dibromo-4-(2,3-dibromopropoxy)phenyl]propane (flame retarder) (**2**), 3,5-di-tert-butyl-4-hydroxytoluene (antioxidant) (**3**), tris(2,4-di-tert-butylphenyl)phosphite (antioxidant) (**4**) 3,9-Bis(octadecyloxy)-2,4,8,10-tetraoxa-3,9-diphosphaspiro[5.5]undecane (antioxidant) (**5**), 2-(3,5-di-tert-amyl-2-hydroxyphenyl)benzotriazole (UV-absorbent) (**6**), 2-hydroxy-4-n-octyloxybenzophenone (UV-absorbent) (**7**), and bis(2,2,6,6-tetramethyl-4-piperidyl)sebacate (photo-stabilizer) (**8**). At room temperature 10 mg of each organic additive (1–8 in Scheme 1) readily resolved in 1 mL of cyclohexanone in a few minutes, and at 150° C the process was more rapid. After cooling the solutions to room temperature almost all the organic additives were still soluble except for DBDPE, which dissolved at a diluted concentration (10 mg/10 mL). The solvency must satisfy the requirements of the isolation process of polyethylene because the amount of additives applied to plastic products are generally less than one percent.

As far as biobased carbon content of precipitates from composites containing petroleum-based fillers indicate (Table 3), collection efficiency of the extraction thimble was adequate to remove fillers. Despite this result, efficiency of the extraction thimble was confirmed via quantitative analysis of calcium derived from calcium carbonate or Gofun (oyster shell) that escaped through the extraction thimble. In the case of the extraction experiment using resin/Gofun composite, calcium was readily detectable (Table 4, Entry 2). The details were not clear. However, a calcium carbonate level of 0.39% in the precipitates was considered a negligible amount for estimation of the biobased carbon content using AMS. The amount induced a slight shift of biobased carbon content (0.05%) because the carbon content of calcium carbonate is lower than that of polyethylene resin (12.0% and 85.6%, respectively). Generally the carbon content of fillers is less than polyethylene resins, except for graphite due to hetero elements in the material. The amount of filler or additive that may shift biobased carbon content by 0.3% (detectable limit of AMS) was calculated at 0.6% for cellulose or starch, and 1.7% for DBDPE. These considerations indicate that this isolation procedure can satisfy the standard pretreatment.

**Table 4:** **Estimation of the collection efficiency of the extraction thimble for calcium carbonate and Gofun (oyster shell) powder**

Entry	Sample (w/w)	Content of calcium carbonate (wt%) ^a^
1	bHDPE/CaCO_3_ (75/25)	0.014
2	bHDPE/Gofun (75/25)	0.390^b^
3	CaCO_3_	0.012^c^
4	Gofun	0.024^b, c^

^a^Measurements of calcium carbonate in recovered precipitates and extraction solutions carried out using ICP.

^b^Content of Gofun powder in the precipitates was calculated on the assumption that Gofun was a pure calcium carbonate.

^c^Content of calcium carbonate in the extraction solvents was calculated based on sample weight.

## Conclusion

Considering the solubility of polyethylene, fillers, and additives applied in polyethylene products, an isolation procedure of resin samples for AMS analysis was examined using a Soxhlet extractor. Solvent-soluble additives and solvent-insoluble fillers in the various model composites were effectively removed to isolate pure resin samples suitable for AMS analysis. Recovery rates of polyethylene from heated solutions of composites (typically 99%), and rejection rates for additives and fillers (>99%) indicate that this procedure is an effective pretreatment for polyethylene products prior to AMS measurements. In addition, results indicated that the biobased synthetic polymer content could be confirmed from the biobased carbon content of resins.
